# CCl_4_ induced genotoxicity and DNA oxidative damages in rats: hepatoprotective effect of *Sonchus arvensis*

**DOI:** 10.1186/1472-6882-14-452

**Published:** 2014-11-21

**Authors:** Huda Mohammad Alkreathy, Rahmat Ali Khan, Muhammad Rashid Khan, Sumaira Sahreen

**Affiliations:** Pharmacology Department, Faculty of Medicine, King Abdulaziz University, Jeddah, Saudia Arabia; Department of Biotechnology, Faculty of Biological Sciences, University of Science and Technology Bannu, Bannu KPK, 28100 Pakistan; Department of Biochemistry, Faculty of Biological Sciences, Quaid-i-Azam University Islamabad, Islamabad, Pakistan; Botanical Sciences Division, Pakistan Museum of Natural History Islamabad, Islamabad, Pakistan

**Keywords:** *Sonchus arvensis*, Carbon tetrachloride, Liver cirrhosis, Lipids peroxidation

## Abstract

**Background:**

*Sonchus arvesis* is traditionally reported in various human ailments including hepatotoxicity in Pakistan. Presently we designed to assess the protective effects of methanolic extract of *Sonchus arvesis* against carbon tetrachloride induced genotoxicity and DNA oxidative damages in hepatic tissues of experimental rats.

**Methods:**

36 male Sprague–Dawley rats were randomly divided into 6 groups to evaluate the hepatoprotective effects of *Sonchus arvensis* against CCl_4_ induced genotoxicity, DNA damages and antioxidant depletion. Rats of normal control group were given free access of food and water *add labitum*. Group II rats received 3 ml/kg of CCl_4_ (30% in olive oil v/v) via the intraperitoneal route twice a week for four weeks. Group III and IV received 1 ml of 100 mg/kg b.w. and 200 mg/kg b.w. SME *via* gavage after 48 h of CCl_4_ treatment whereas group V was given 1 ml of silymarin (100 mg/kg b.w.) after 48 h of CCl_4_ treatment. Group VI only received 200 mg/kg b.w. SME. Protective effects of SME were checked by measuring serum markers, activities of antioxidant enzymes, genotoxicity and DNA dmages.

**Results:**

Results of the present study showed that treatment of SME reversed the activities of serum marker enzymes and cholesterol profile as depleted with CCl_4_ treatment. Activities of endogenous antioxidant enzymes of liver tissue homogenate; catalase (CAT), superoxide dismutase (SOD), glutathione peroxidase (GSHpx), glutathione-S-transferase (GST) and glutathione reductase (GSR) were reduced with administration of CCl_4_, which were returned to the control level with SME treatment. CCl_4_-induced hepatic cirrhosis decreased hepatic glutathione (GSH) and increased lipid peroxidative products (TBARS), were normalized by treatment with SME. Moreover, administration of CCl_4_ caused genotoxicity and DNA fragmentation which were significantly restored towards the normal level with SME.

**Conclusion:**

These results reveal that treatment of SME may be useful in the prevention of hepatic stress.

## Background

Carbon tetrachloride (CCl_4_), a clear, colorless, volatile, heavy and nonflammable industrial liquid, widely used to inducede free radical toxicity in various tissues of experimental animals such as liver, kidneys, heart, lung, testis, brain and blood [1]. CCl_4_ is converted through hepatic microsomal cytochrome P450 into trichloromethyl-free radical (∙CCl_3_ or ∙CCl_3_OO) [2] which in turn, initiate lipid peroxidation process [3, 4]. The most widely accepted mechanism of CCl_4_ induced hepatotoxicity is the formation of free radicals which is a rate limiting process in tissue peroxidative damage [5, 6]. This free radical and related reactive species may cause oxidative stress, which produces major interconnected changes of cellular metabolism, increases the serum marker enzymes, DNA fragmentation, and destruction of the cells by lipid peroxidation [7]. The accumulation of lipid peroxides introduces hydrophophilic moieties and alters membrane permeability and cell function which causes the loss of hepatic integrity and depressed hepatic function resulting in hepatotoxicity and congestive hepatic failure [8]. To protect the body from such deleterious effects of free radicals, several endogenous enzymatic and non enzymatic systems are provided, but when the formation of free radicals is excessive, additional protective mechanisms of dietary antioxidants may be of a great importance [9]. Maintaining the balance between reactive oxygen species and natural antioxidants is therefore crucial, and could serve as a major mechanism in preventing damage by oxidative stress induced by toxic agents. Cooperative defense systems that protect the body from free radical damage include the antioxidant nutrients and enzymes [10]. Antioxidant and radical scavengers have been used to study the mechanism of CCl_4_ toxicity as well as to protect tissue cells from CCl_4_ induced damage by breaking the chain of lipid peroxidation [11]. Numerous studies have shown that horticultural crops and fruits are sources of diverse antioxidant properties, which can protect body against CCl_4,_ induced oxidative stress [12]. *Sonchus arvensis* is traditionally used in the treatment of kidney stone, gallstone, dysentri, haemorrhoid, gout arthritis, appendicitis, mastitis, hypertension, burn wound, and bruises. The present study was therefore designed to investigate the protective effect of *Sonchus arvensis* (SME) against CCl_4_ induced hepatotoxicity in rats.

## Methods

### Drugs and chemicals

Reduced glutathione (GSH), oxidized glutathione (GSSG), glutathione reductase, gamma-glutamyl p-nitroanilide, glycylglycine, bovine serum albumin (BSA), 1,2-dithio-bis nitro benzoic acid (DTNB), 1-chloro-2,4-dinitrobenzene (CDNB), reduced nicotinamide adenine dinucleotide phosphate (NADPH), CCl_4_, flavine adenine dinucleotide (FAD), glucose-6-phosphate, Tween-20, 2,6-dichlorophenolindophenol, thiobarbituric acid (TBA), picric acid, sodium tungstate, sodium hydroxide, trichloroacetic acid (TCA) and perchloric acid (PCA) were purchased from Sigma Chemicals Co. USA.

### Animals and treatment

Six weeks old, 36 rats (200–210 g) were provided by National Institute of Health Islamabad and were kept in ordinary cages at room temperature of 25 ± 3°C with a 12 h dark/light cycles. They have free access to standard laboratory feed and water, according to the study protocol approved by Ethical Committee of University of Science and Technology Bannu, KPK, Pakistan. To study the hepatoprotective effects of SME, rats were equally divided into 6 groups (six rats). SME was administered after 48 h of CCl_4_ treatment for four weeks.

Group I: Control; standard diet and water

Group II: CCl_4_ (3 ml/kg b.w. i.p.)

Group III: CCl_4_ (3 ml/kg b.w. i.p.) + SME (100 mg/kg b.w. orally)

Group IV: CCl_4_ (3 ml/kg b.w. i.p.) + SME (200 mg/kg b.w. orally)

Group V: CCl_4_ (3 ml/kg b.w. i.p.) + Silymarin (100 mg/kg b.w. orally)

Group VI: SME (200 mg/kg b.w. orally) alone

After 24 h of the last treatment, all the animals were weighted, sacrificed, collected the blood while liver were removed, weighted and perfuse in ice-cold saline solution. Liver tissue was treated with liquid nitrogen for further studies.

### Assessment of hepatotoxicity

Liver marker enzymes (alanine aminotransferase (ALT), aspartate aminotransferase (AST), alkaline phosphatase (ALP), gamma glutamyl transpeptidase (γ-GT), lipid profile (total cholesterol, low-density lipoprotein (LDL), high-density lipoprotein (HDL) and triglyceride were estimated by using standard AMP diagnostic kits (Stattogger Strasse 31b 8045 Graz, Austria).

### Assessment of oxidative stress

Hepatic tissue were homogenized in 10 volume of 100 mmol KH_2_PO_4_ buffer containing 1 mmol EDTA (pH 7.4) and centrifuged at 12,000 × g for 30 min at 4°C. The supernatant was collected and used for the assessment of antioxidant enzymes. Protein concentration in the supernatant of liver tissue homogenate was determined using crystalline BSA as standard. The entire chemicals used in enzymatic analysis were purchased form sigma.

### Catalase assay (CAT)

CAT activities were determined by the method of Chance and Maehly [13] with some modifications. The reaction solution of CAT activities contained: 2.5 ml of 50 mmol phosphate buffer (pH 5.0), 0.4 ml of 5.9 mmol H_2_O_2_ and 0.1 ml enzyme extract. Changes in absorbance of the reaction solution at 240 nm were determined after one minute. One unit of CAT activity was defined as an absorbance change of 0.01 as units/min.

### Superoxide dismutase assay (SOD)

SOD activity of liver tissue was estimated by the method of Kakkar et al. [14]. Reaction mixture of this method contained: 0.1 ml of phenazine methosulphate (186 μmol), 1.2 ml of sodium pyrophosphate buffer (0.052 mmol; pH 7.0), 0.3 ml of supernatant after centrifugation (1500 × g for 10 min followed by 10000 × g for 15 min) of homogenate was added to the reaction mixture. Enzyme reaction was initiated by adding 0.2 ml of NADH (780 μmol) and stopped after 1 min by adding 1 ml of glacial acetic acid. Amount of chromogen formed was measured by recording color intensity at 560 nm. Results were expressed in units/mg protein.

### Glutathione-S-transferase assay (GST)

Glutathione-S-transferase activity was assayed by the method of Habig et al. [15]. The reaction mixture consisted of 1.475 ml phosphate buffer (0.1 mol, pH 6.5), 0.2 ml reduced glutathione (1 mmol), 0.025 ml (CDNB) (1 mmol) and 0.3 ml of homogenate in a total volume of 2.0 ml. The changes in the absorbance were recorded at 340 nm and enzymes activity was calculated as nmol CDNB conjugate formed/min/mg protein using a molar extinction coefficient of 9.6 × 10^3^ M^-1^ cm^-1^.

### Glutathione reductase assay (GSR)

Glutathione reductase activity was determined by method of Carlberg and Mannervik [16]. The reaction mixture consisted of 1.65 ml phosphate buffer: (0.1 mol; pH 7.6), 0.1 ml EDTA (0.5 mmol), 0.05 ml oxidized glutathione (1 mmol), 0.1 ml NADPH (0.1 mmol) and 0.1 ml of homogenate in a total volume of 2 ml. Enzyme activity was quantitated at 25°C by measuring disappearance of NADPH at 340 nm and was calculated as nmol NADPH oxidized/min/mg protein using molar extinction coefficient of 6.22 × 10^3^ M^-1^ cm^-1^.

### Glutathione peroxidase assay (GSH-Px)

Glutathione peroxidase activity was assayed by the method of Mohandas et al. [17]. The reaction mixture consisted of 1.49 ml phosphate buffer (0.1 mol; pH 7.4), 0.1 ml EDTA (1 mmol), 0.1 ml sodium azide (1 mmol), 0.05 ml glutathione reductase (1 IU/ml), 0.05 ml GSH (1 mmol), 0.1 ml NADPH (0.2 mmol), 0.01 ml H_2_O_2_ (0.25 mmol) and 0.1 ml of homogenate in a total volume of 2 ml. The disappearance of NADPH at 340 nm was recorded at 25°C. Enzyme activity was calculated as nmol NADPH oxidized/min/mg protein using molar extinction coefficient of 6.22 × 10^3^ M^-1^ cm^-1^.

### Reduced glutathione assay (GSH)

Reduced glutathione was estimated by the method of Jollow et al. [18]. 1.0 ml sample of homogenate was precipitated with 1.0 ml of (4%) sulfosalicylic acid. The samples were kept at 4°C for 1 h and then centrifuged at 1200 × g for 20 min at 4°C. The total volume of 3.0 ml assay mixture contained 0.1 ml filtered aliquot, 2.7 ml phosphate buffer (0.1 mol; pH 7.4) and 0.2 ml DTNB (100 mmol). The yellow color developed was read immediately at 412 nm on a SmartSpecTM plus Spectrophotometer. It was expressed as μmol GSH/g tissue.

### Estimation of lipid peroxidation assay (TBARS)

The assay for lipid peroxidation was carried out by the modified method of Iqbal et al. [19]. The reaction mixture in a total volume of 1.0 ml contained 0.58 ml phosphate buffer (0.1 mol; pH 7.4), 0.2 ml homogenate sample, 0.2 ml ascorbic acid (100 mmol), and 0.02 ml ferric chloride (100 mmol). The reaction mixture was incubated at 37°C in a shaking water bath for 1 h. The reaction was stopped by addition of 1.0 ml 10% trichloroacetic acid. Following addition of 1.0 ml 0.67% thiobarbituric acid, all the tubes were placed in boiling water bath for 20 min and then shifted to crushed ice-bath before centrifuging at 2500 × g for 10 min. The amount of TBARS formed in each of the samples was assessed by measuring optical density of the supernatant at 535 nm using spectrophotometer against a reagent blank. The results were expressed as nmol TBARS/min/mg tissue at 37°C using molar extinction coefficient of 1.56 × 10^5^ M^-1^ cm^-1^.

### DNA fragmentation% assay

DNA fragmentation% assay was conducted using the procedure of Wu et al. [20] with some modifications. The tissue (50 mg) was homogenized in 10 volumes of a TE solution pH 8.0 (5 mmol Tris–HCl, 20 mmol EDTA) and 0.2% triton X-100. 1.0 ml aliquot of each sample was centrifuged at 27,000 × g for 20 min to separate the intact chromatin (pellet, B) from the fragmented DNA (supernatant, T). The pellet and supernatant fractions were assayed for DNA content using a freshly prepared DPA (Diphenylamine) solution for reaction. Optical density was read at 620 nm at (SmartSpecTM Plus Spectrophotometer catalog # 170–2525) spectrophotometer. The results were expressed as amount of % fragmented DNA by the following formula;


### DNA ladder assay

DNA was isolated by using the methods of Wu et al. [20] to estimate DNA damages. 5 μg DNA of rats were separately loaded in 1.5% agarose gel containing 1.0 μg/ml ethidium bromide including DNA standards (0.5 μg per well). Electrophoresis was performed for 45 min at 100 Volt. After electrophoresis gel was studied under gel doc system and was photographed through digital camera.

### AgNORs count

Silver staining technique was used according to the Trere et al. [21]. The AgNORs technique was performed on dried slides as follows; unstained fixed slides were dewaxed by dipping for 3 minutes in xylene. After complete removal of wax the slides were hydrated in decrease ethanol concentration (90, 70 and 50%) and washed in distilled water for 10 min and dried in an oven. After drying slides were treated with one drop of colloidal solution (2% gelatin and 1% formic acid) and two drops of 50% AgNO_3_ solution onto the slide and incubated at 35°C for about 8–12 min. The progressive staining was followed under microscope to get golden colored nuclei and brown/black NORs. Then, the slide was washed in distilled water, treated for 1 min with 1% sodium thiosulphate at room temperature to stop the reaction, and washed in tap water. The cells were examined under light microscope at 100 × magnification and number of AgNORs was counted per cell.

### Statistical analysis

To determine the treatment effects, one-way analysis of variance was carried by computer software SPSS 13.0. Level of significance among the various treatments was determined by LSD at 0.05% and 0.01% level of probability.

## Results

Treatment of CCl_4_ specifically targets the hepatocytes. CCl_4_ induced oxidative stress cause lesions in liver along with changes in the liver marker enzymes, biochemical markers and antioxidant defense enzymes and chemicals. The results obtained with CCl_4_ treatment and changes induced with SME are given below.

### Body weight, liver weight

Treatment of CCl_4_ caused significant reduction (*P* < 0.01) in body weight while increased the absolute liver and relative liver weight comparatively to control group; were significantly *(P <* 0.01*)* restored with treatment of 10 mg/kg b.w., and 200 mg/kg b.w., SME (Table 1).

**Table 1:** **Effect of SME on body weight, liver weight and relative liver weight**

Treatment	% Increase in body weight	Liver weight (g)	Relative liver weight (% to body weight)
Control	28.90 ± 2.17^++^	7.0 ± 0.83^++^	0.07 ± 0.002^++^
3 ml/kg CCl_4_	19.57 ± 3.02**	9.6 ± 0.89**	0.96 ± 0.006**
100 mg/kg SME + CCl_4_	25.28 ± 1.51^++^	7.48 ± 0.70^++^	0.074 ± 0.002^++^
200 mg/kg SME + CCl_4_	27.14 ± 2.63^++^	7.14 ± 0.53^++^	0.071 ± 0.003^++^
100 mg/kg sylimarin + CCl_4_	27.01 ± 1.26^++^	7.22 ± 0.75^++^	0.072 ± 0.001^++^
200 mg/kg SME alone	29.02 ± 2.49^++^	7.03 ± 0.67^++^	0.070 ± 0.006^++^

Mean ± SE (n = 6 number).

**indicate significance from the control group at P < 0.05 and P < 0.01 probability level.

^++^indicate significance from the CCl_4_ group at P < 0.05 and P < 0.01 probability level.

### Lipids profile

Administration of CCl_4_ increased triglycerides, total cholesterol, LDL cholesterol while decreased the HDL cholesterol as shown in Table 2. Reduction of HDL cholesterol was significantly *(P < 0.01)* enhanced by SME while triglycerides, total cholesterol and LDL-cholesterol concentration was appreciably *(P <* 0.01*)* augmented to compensate the CCl_4_ group.

**Table 2:** **Effect of SME on liver markers enzymes**

Treatment	ALT(U/L)	AST(U/L)	ALP(U/L)	γ-GT(nM/min /mg protein)
Control	45.8 ± 3.2^++^	53.8 ± 3.4^++^	148 ± 5.9^++^	105.5 ± 2.2^++^
3 ml/kg CCl_4_	102 ± 4.2**	94.0 ± 4.7**	340.3 ± 6.9**	154.3 ± 3.2**
100 mg/kg SME + CCl_4_	68 ± 3.8^++^	62.3 ± 4.9^++^	207.5 ± 4.9^++^	121.3 ± 3.4^++^
200 mg/kg SME + CCl_4_	91 ± 2.2^++^	56 ± 4.1^++^	167.7 ± 5.7^++^	108 ± 2.7^++^
100 mg/kg sylimarin + CCl_4_	89 ± 1.5^++^	57.5 ± 2.0^++^	157.8 ± 3.9^++^	110 ± 3.5^++^
200 mg/kg SME alone	97 ± 2.4^++^	49.5 ± 3.6^++^	145.3 ± 5.1^++^	103 ± 2.7^++^

Mean ± SE (n = 6 number).

**indicate significance from the control group at *P < 0.05* and *P < 0.01* probability level.

^++^indicate significance from the CCl_4_ group at *P < 0.05* and *P < 0.01* probability level.

### Genotoxicity studies

**Figure 1:**
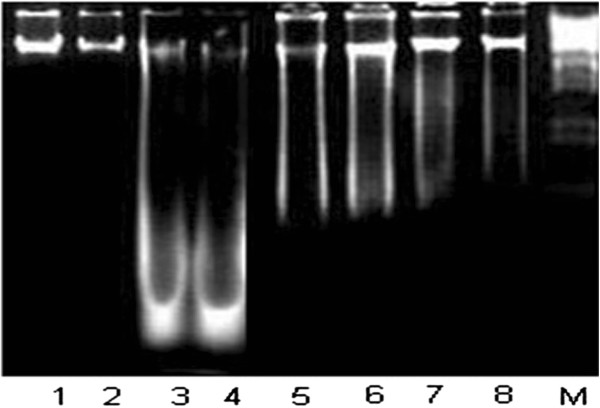
**Protective effects of SME on DNA; Lane 1–2 (control), 3–4 (CCl4 treated rats), 5,6 (CCl4 + 100 mg/kg b.w. SME), 7,8 (CCl4 + 200 mg/kg b.w. SME).**

Exposure of CCl_4_ elicited the hepatic DNA damages (%fragmentation), number of AgNORs/cell. Treatment of rats with 100 mg/kg b.w. and 200 mg/kg b.w. SME restored the level of these markers (Table 3). DNA ladder assay showed conformity to the DNA fragmentation assay (Figure 1).

**Table 3:** **Effect of SME on liver markers enzymes**

Treatment	AgNORS (NORs/cell)	%DNA fragmentation
Control	2.0 ± 0.33^++^	5.33 ± 2.46^++^
3 ml/kg CCl_4_	6.4 ± .29**	22.50 ± 3.68**
100 mg/kg SME + CCl_4_	3.1 ± 0.35*^++^	5.00 ± 1.83^++^
200 mg/kg SME + CCl_4_	3.5 ± 0.18**^++^	6.67 ± 2.08^++^
100 mg/kg sylimarin + CCl_4_	2.14 ± 0.23^++^	5.67 ± 3.12^++^
200 mg/kg SME alone	1.9 ± 0.17**^++^	4.67 ± 2.23^++^

Mean ± SE (n = 6 number).

**indicate significance from the control group at *P < 0.05* and *P < 0.01* probability level.

^++^indicate significance from the CCl_4_ group at *P < 0.05* and *P < 0.01* probability level.

### Liver function profile

Administration of CCl_4_ markedly increased *(P < 0.01)* the activity of liver serum marker enzymes such as AST, ALT, ALP and γ-GT as compared with the control group. Elevations in the secretion of these enzymes were significantly decreased *(P < 0.01)* by 100 mg/kg b.w. and 200 mg/kg b.w. SME as compared with the CCl_4_ group are shown in Table 4.

**Table 4:** **Effect of SME on liver cholesterol profile**

Treatment	TG (mg/dl)	TC (mg/dl)	HDL (mg/dl)	LDL (mg/dl)
Control	7.8 ± 0.45^++^	6.1 ± 0.25^++^	3.6 ± 0.21^++^	2.48 ± 0.32^++^
3 ml/kg CCl_4_	11.3 ± 0.58**	11.2 ± 0.23**	2.8 ± 0.18**	8.4 ± 0.17**
100 mg/kg SME + CCl_4_	8.5 ± 0.44^++^	5.7 ± 0.20**^++^	3.2 ± 0.23^++^	2.52 ± 0.28^++^
200 mg/kg SME + CCl_4_	9 ± 0.41**^++^	7.7 ± 0.21**^++^	3.08 ± 0.09^++^	4..2 ± 0.21**^++^
100 mg/kg sylimarin + CCl_4_	8.3 ± 0.18^++^	6.4 ± 0.27^++^	3.5 ± 0.20^++^	2..53 ± 0.35^++^
200 mg/kg SME alone	7.2 ± 0.44^++^	5.7 ± 0.19^++^	3.7 ± 0.21^++^	2.21 ± 0.31^++^

Mean ± SE (n = 6 number).

**indicate significance from the control group at *P < 0.05* and *P < 0.01* probability level.

^++^indicate significance from the CCl_4_ group at *P < 0.05* and *P < 0.01* probability level.

### Assessment of oxidative stress

CCl_4_ treatment in rats significantly decreased *(P <* 0.01*)* the activity of CAT, SOD, GST, GSH-Px, GSR, GSH while increased TBARS contents. The increase of lipid peroxidation caused; reduction in the activities of antioxidant enzymes and glutathione (GSH) contents were markedly attenuated *(P <* 0.01*)* by administration of 100 mg/kg and 200 mg/kg b.w. of SME in intoxicated rats (Table 5).

**Table 5:** **Effect of SME on antioxidant profile**

Treatment	CAT (U/min )	SOD (U/mg protein)	GSH-Px nM/min/mg protein	GSH (μM /min/mg protein)	GSR nM/min/mg protein)	TBARS(nM /min/mg protein)
Control	6.0 ± 0.5^++^	18.7 ± 2.8^++^	64.7 ± 3.9^++^	2.12 ± 0. 2^++^	121.7 ± 6.4^++^	29.3 ± 1.2^++^
3 ml/kg CCl_4_	2.9 ± 0.6**	9.9 ± 0.7**	34.2 ± 6.3**	1.03 ± 0.3**	67.3 ± 3.5**	53.17 ± 1.2**
100 mg/kg SME + CCl_4_	5.0 ± 0.7^++^	16.5 ± 0.7^++^	52.4 ± 7.8^++^	1.90 ± 0.1^++^	111.2 ± 12.4^++^	38.7 ± 2.6^++^
200 mg/kg SME + CCl_4_	5.8 ± 0.9^++^	17.5 ± 0.8^++^	62.7 ± 5.6	2.03 ± .07	122.33 ± 5.28^++^	31.17 ± 1.4^++^
100 mg/kg sylimarin + CCl_4_	5.7 ± 0.5^++^	19.4 ± 0.3^++^	60.2 ± 5.3^++^	2.17 ± 0.04^++^	115.3 ± 9.14^+^	30.0 ± 2.7^++^
200 mg/kg SME alone	5.9 ± 0.6^++^	20.9 ± 0.5^++^	66.8 ± 3.3^++^	2.09 ± 0.2^++^	120.2 ± 6.3^++^	31.2 ± 2.7^++^

Mean ± SE (n = 6 number).

**indicate significance from the control group at *P < 0.01* probability level.

^++^indicate significance from the CCl_4_ group at *P < 0.01* probability level.

## Discussion

Metabolism of various metabolites and exogenous toxic chemicals (pesticides, drugs, metals), are takes place inside the hepatic tissue causes the formation of free radicals which may be extensively toxic than the parent compound. CCl_4_, an extensively studied hepatotoxin is converted into its metabolites such as CCl_3_ radicals which are involved in the liver pathogenesis including cirrhosis, genotoxicity of hepatic tissue and hepatic carcinoma [8]. Our present results showed that exposure of rats to CCl_4_ caused significant increase in the secretion of ALT, AST, ALP, γ-GT and cholesterol profile due to hepatic injuries caused by their free radicals [22]. Co-administration of 100 mg/kg and 200 mg/kg b.wSME significantly improved the pathogenesis of liver, might be due to the presence of poly phenolic constituent as was reported by Xiao et al. [23]. SME might have the ability to chealat free radical which in turn lowering serum cholesterol, triglycerides and lipid peroxide were reported in other investigations while working on hepatoprotective effects of plant extract against CCl_4_ induced hepatic injury in rats [24, 25]. Super oxide dismutase and catalase are the main antioxidant enzymes which play an important role in oxidative dysfunction against free radicals induced oxidative stress. Results of our investigation showed that CCl_4_ administration in rats result in depletion of antioxidant activities of SOD and CAT, which is in close relationship with other reports [26, 27] and have an agreement with investigation following CCl_4_ intoxication [28]. GSH is an important protein thiol which coordinates body defense system against oxidative stress. GSH effectively scavenge free radicals and other reactive oxygen species (e.g., hydroxyl radical, lipid peroxy radical, peroxy nitrite and H_2_O_2_) directly or through GSHpx, GST and GSR [29]. Present study revealed that induction of CCl_4_ caused significant reduction in GSH contents as well as significant depletion in the activity of phase II metabolizing enzymes; GSH-px, GST and GSR [30]. Co-treatment of SME in rats markedly improved the activity of metabolizing enzymes as mentioned in literature. TBARS is a major reactive aldehyde resulting during the peroxidation of polyunsaturated fatty acids (PUFA) a useful indicator of oxidative damages [31–34]. Results revealed that 100 mg/kg and 200 mg/kg b.w. SME significantly improved lipid peroxidation products as was altered by treatment of CCl_4_ in rats, which has been well documented [35]. According to Marnett [36] the product of lipid peroxidation react with DNA to form adducts MIG, the mutagenic pirimedopurinone adduct of deoxyguanosine. Like other macromolecules such as lipids and proteins, nucleic acids are also attacked by free radicals to cause oxidative DNA damage. In the present study, carbon tetrachloride degrades the DNA of liver tissue of rats by generating free radicals. On the other hand, co-treatment of SME appreciably reduced the DNA fragmentation% which also exposed by DNA ladder assay banding pattern. Similar results were reported by Murugesan et al. [37] while studying the protective effects of *Kombucha tea* against CCl_4_ induced oxidative stress in kidneys of rats.

## Conclusion

These results demonstrate that administration of SME may be useful in the treatment and prevention of hepatic genotoxicity and oxidative stress.
